# Particle Morphology Analysis of Biomass Material Based on Improved Image Processing Method

**DOI:** 10.1155/2017/5840690

**Published:** 2017-02-19

**Authors:** Zhaolin Lu, Xiaojuan Hu, Yao Lu

**Affiliations:** ^1^Advanced Analysis and Computation Center, China University of Mining and Technology, Xuzhou 221116, China; ^2^School of Sciences, China University of Mining and Technology, Xuzhou 221116, China

## Abstract

Particle morphology, including size and shape, is an important factor that significantly influences the physical and chemical properties of biomass material. Based on image processing technology, a method was developed to process sample images, measure particle dimensions, and analyse the particle size and shape distributions of knife-milled wheat straw, which had been preclassified into five nominal size groups using mechanical sieving approach. Considering the great variation of particle size from micrometer to millimeter, the powders greater than 250 *μ*m were photographed by a flatbed scanner without zoom function, and the others were photographed using a scanning electron microscopy (SEM) with high-image resolution. Actual imaging tests confirmed the excellent effect of backscattered electron (BSE) imaging mode of SEM. Particle aggregation is an important factor that affects the recognition accuracy of the image processing method. In sample preparation, the singulated arrangement and ultrasonic dispersion methods were used to separate powders into particles that were larger and smaller than the nominal size of 250 *μ*m. In addition, an image segmentation algorithm based on particle geometrical information was proposed to recognise the finer clustered powders. Experimental results demonstrated that the improved image processing method was suitable to analyse the particle size and shape distributions of ground biomass materials and solve the size inconsistencies in sieving analysis.

## 1. Introduction

With the depletion of fossil fuels and their corresponding undesirable effects on the environment, biomass utilization has received increased attention. Because of its renewability and abundance, biomass is considered to be one of the most promising resources, which can be converted into gaseous [1], liquid [2], and solid fuels [3] and other chemical raw materials or products [4–6].

Morphological characteristics of particles, including size distribution and shape factor, are important in these biomass applications [7–10]. Bridgeman et al. [11] studied the influence of particle size on the analytical and chemical properties of switchgrass and reed canary. Hendriks and Zeeman [12] found that a decreasing of biomass particle size involves higher hydrolysis yields of the lignocellulose. Gil et al. [13] also observed the underlying mechanism that govern the handling behavior for poplar and corn stover is partially influenced by the particle size and shape.

Size reduction, known as grinding process, is a critical procedure because it changes the particle size and shape of biomass. The surface area of ground particles increases the number of contact points for chemical reactions, which improves the energy conversion efficiency of biomass [14–18]. Mechanical sieving is the most standard method adopted by the American Society of Agricultural and Biological Engineers (ASABE) for particle size analysis of ground biomass. In practice, measured geometric mean length of biomass particles using sieve analysis is less than the actual size of the particles [19–21]. Other existing methods of PSD measurement use the principles of light scattering, acoustic spectroscopy and laser diffraction. Such methods often assume the particles to be spherical, which is not always the predominant case with ground biomass materials [22].

Image processing technology is considered as an alternative for mechanical sieving in PSD and shape identification analysis [23–26]. The two basic steps of image-based particle size and shape analysis are image acquisition and processing. Devices for image acquisition include flatbed scanners [27] and digital cameras [28], which are limited by image resolution and depth of field, and are suitable only for photographing millimeter-sized or larger particles. By means of high magnification, scanning electron microscopy (SEM) has been widely applied in analysing micron-sized or smaller particles, such as wood dust from furniture manufacturing [29] and even atmospheric particles in industrial areas [30]. Owing to their built-in image analysis functions, most image processing algorithms reported in the literature use proprietary software, such as ImageJ [31]. To obtain special dimensional information of powders, some image analysis algorithms may require a flexible programming language environment such as Matlab (MathWorks, USA) with specialized image processing toolboxes [32].

Although particle morphology analysis based on image processing method has been the subject of many studies, the implementation of experiments and algorithms as important steps in the analysis procedure have received less attention. In this study, a series of improvements were conducted on sample preparation, image acquisition, and processing algorithms. The particulate sample was first divided into five groups by mechanical sieving approach to reduce wider size ranges. Considering the great variation of particle size from micrometer to millimeter, we utilized two acquisition units with different resolutions to photograph the particulate sample. Particle aggregation is an important factor that influences the recognition accuracy of the image processing method. In terms of sample preparation, the singulated arrangement and ultrasonic dispersion methods were used to disperse the powders. In addition, an image segmentation algorithm based on particle geometrical information was proposed to recognise the finer clustered powders.

## 2. Materials and Methods

### 2.1. Materials

Wheat straw is an herbaceous resource for the bioethanol production [33]. As a traditional crop with one of the highest yields, wheat straw obtained from the suburb of Xuzhou in China was chosen as the test sample in this study. Before grinding, wheat straw should be pretreated in size; the average length of the wheat straw materials was approximately 15 mm. Then, the samples fed into the laboratory-scale knife mill were placed in an oven at 103°C for 24 h before grinding, thereby reducing its wet basis moisture content from 27% to 5%, as measured by an infrared moisture meter.

### 2.2. Grinding and Sieving Stage

**Figure 1:**
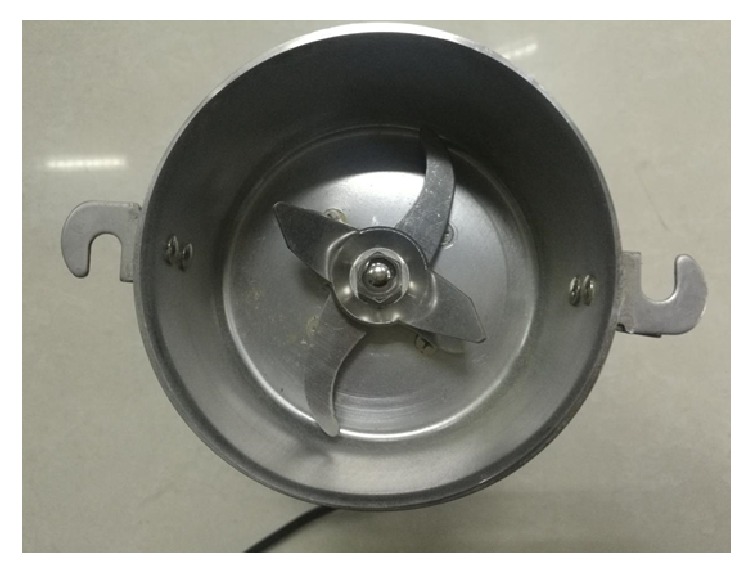
Blades of the knife mill.

A commercially available knife mill (FW177, Laibu) operating at 28000 rpm was used to grind wheat straw based on the shearing action of the knife blades [34]. The corresponding grinding blades of the knife mill are shown in Figure 1.

Approximately 50 g of dried wheat straw samples, weighed by an electronic analytical balance (±0.001 mg accuracy, Sartorius), were ground using the knife mill. The ground materials were semiclassified using a mechanical sieving instrument (AS 200 Control, Retsch) with four sieves of mesh sizes 75, 250, 500, and 1000 *µ*m. Commonly, sieving procedure is used to obtain the PSD, but for this study, is used to classify the particles that later will be photographed and analysed for the particle morphology analysis. After the shaking was completed, the stack was removed from the sieves and carefully weighed, and the mass of each sieve was recorded with its retained powders.

Based on pretests, the grinding and corresponding sieving times were determined to be both 15 min, which were the times at which the mass of the test sieves did not change by more than 5% of the previous mass on the sieves [35]. Classification by sieving reduced the wide size range of ground particles, not only to precisely photograph the powders using an image acquisition device but also to understand the size range of particles generated by the knife mill. After grinding and sieving, the samples were sorted into five size fractions: >1000, 500–1000, 250–500, 75–250, and <75 *μ*m.

### 2.3. Sample Preparation and Image Acquisition

Clear contrast between particles and background is essential to effectively recognise the particles. Igathinathane et al. [23] proposed that particles should be well spread in a singulated arrangement, where a thin layer of samples is laid in a manner that particles do not touch or overlap one another. However, when particles are considerably small, avoiding agglomeration caused by van der Waals force from a singulated arrangement is difficult. Thus, to address the wider size range and avoid particle agglomeration, an experimental method, including sample preparation and image acquisition was improved.

In this study, the singulated arrangement based on manual separation was used to spread larger particles, such as size ranges of >1000, 500–1000, and 250–500 *μ*m. Then, the well-scattered samples were photographed by a flatbed scanner (Laser Jet M1213nf, HP) with a resolution of 600 dpi, which corresponded to a constant scale factor of 42.3 *μ*m pixel^−1^. Preliminary tests verified that the flatbed scanner without zoom function was unsuitable to photograph particles that were less than nominal size of 250 *μ*m.

**Figure 2:**
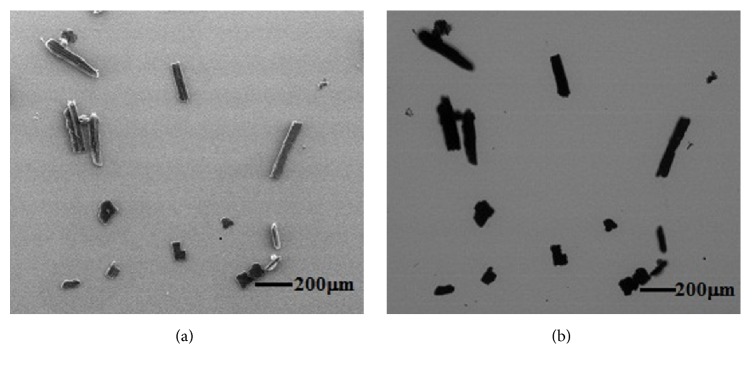
Particle images acquired by SEM: (a) SE image; (b) BSE image.

Ultrasonic dispersion method was used to scatter these particles, thereby weakening the particle agglomeration of size ranges of 75–250 and <75 *μ*m. Approximately 5 mg of each size range was suspended in 10 ml of anhydrous ethanol. Then, the suspension was stirred continuously for 10 min in an ultrasonic cleaner to avoid particle aggregation. About 0.5 ml of the aforementioned suspension was dropped on a mica substrate, which was flat enough to highlight the contrast between the particles and image background. After air drying, the samples along with substrate were placed in an SEM chamber (Quanta 250, FEI), which had a huge advantage over field of view and depth of field relative to an optical microscope. SEM has two imaging modes, namely, secondary electron (SE) and backscattered electron (BSE).  Figure 2(a) shows that particles are inhomogeneous at grey level caused by Edge Effect which is due to the enhanced emission of electrons from edges within the specimen. These inhomogeneities of particles are unfavourable for segmenting particles from the background. The grey level of the BSE image was determined by the average atomic number of material. Biomass materials are classified as organic hydrocarbons with average atomic number smaller than that of mica substrate.  Figure 2(b) shows the clear contrast between particles and background for the BSE image. In this test, the BSE imaging mode of SEM was used to acquire particle images, and the image magnification for size ranges of 75–250 and <75 *μ*m was set to 200x and 300x, respectively; thus, the corresponding scale factors were 1.46 *μ*m pixel^−1^ and 0.971 *μ*m pixel^−1^.

**Figure 3:**
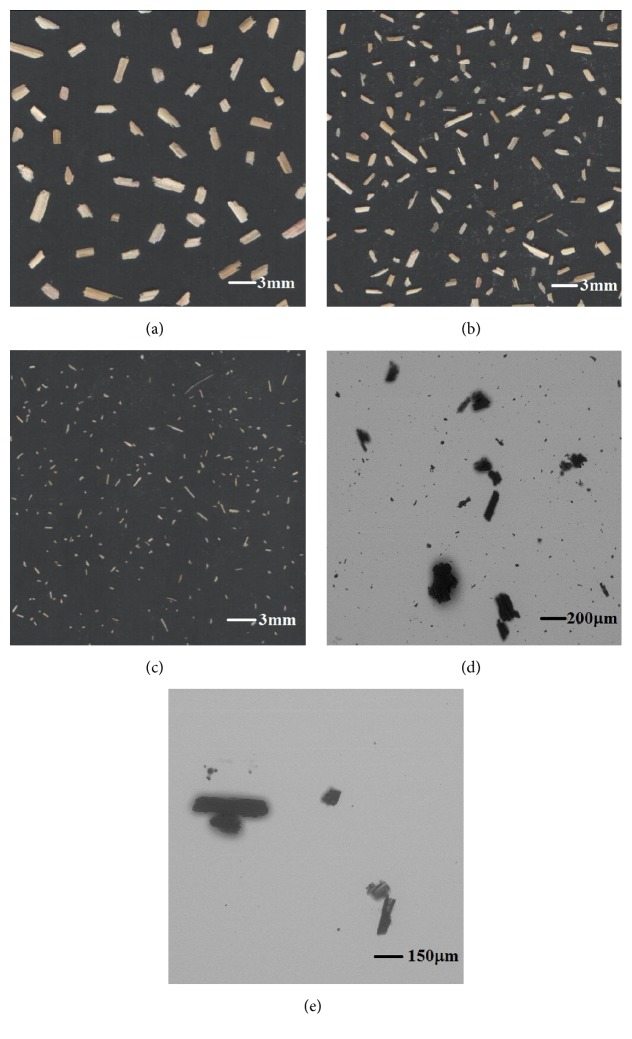
Sample images of ground wheat straw particles from five size ranges: (a) >1000 *μ*m, (b) 500–1000 *μ*m, (c) 250–500 *μ*m, (d) 75–250 *μ*m, and (e) <75 *μ*m.

Based on the preceding analysis, the dispersive particle images of each size range were acquired by two imaging devices. For each size ranges, the minimum particle area to be taken into consideration was set to 4 pixels to ignore ultrafine particles. Figures 3(a)–3(c) show the larger particle images obtained by the flatbed scanner, and Figures 3(d)-3(e) show images photographed by SEM.

### 2.4. Image Processing and Dimension Measurement

**Figure 4:**
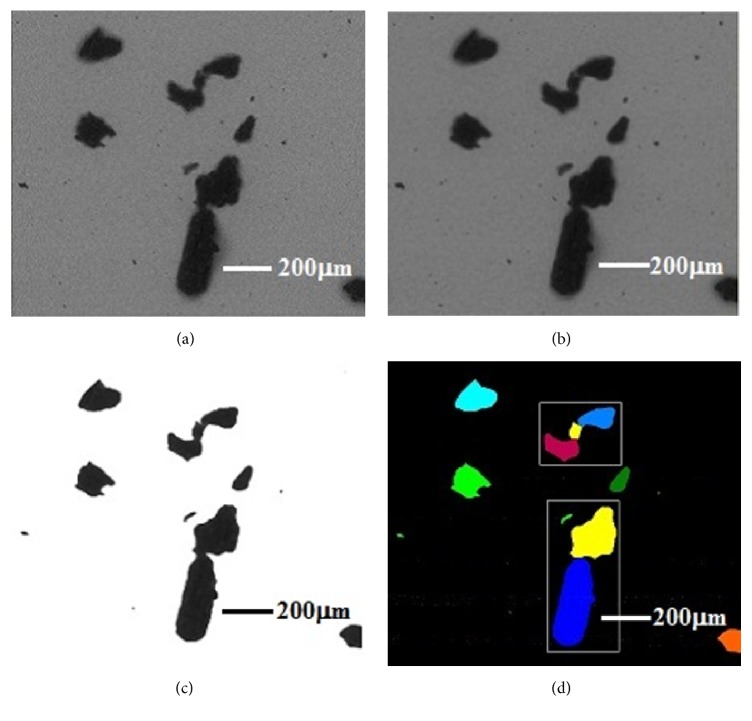
Processing results corresponding to each step.

Image processing, an essential preliminary step in most automated particle morphology analysis, is used to subdivide an image into constituent regions. The accuracy of this process determines the eventual accuracy of PSD. An image processing and dimension measurement method was proposed to recognise biomass particles of 75–250 *μ*m in a BSE image following the steps shown in Figure 4.

*Step  1*. The noise from the original image was removed. For convenience, a Gaussian filter, a simple and quick denoising method, was selected to denoise the original image. Figures 4(a) and 4(b) show the original and denoised images, respectively.

*Step  2*. The denoised image was converted to binary image using a single global threshold value of the entire image. Otsu's method was used for binarisation, which was deemed suitable if all particles had obvious intensity difference from the background. Adopting mica as the sample substrate and BSE imaging mode emphasised the grey level difference and reduced the burden of threshold setting. The output of this procedure was a binary image shown in Figure 4(c).

*Step  3*. The holes of recognised particles were filled and the touching particles were separated. Holes were generated after binarisation for large particles because of their high grey level difference. The distance between the location of the hole and edge of the particles was used as basis to determine whether the holes needed to be filled or not. In addition, the touching particles were falsely recognised as one particle because of the agglomeration effect. Basic morphological operators, such as erosion, dilation, opening, and closing, cause changes in the size of particles in the binary images, which is not allowed for particle morphology analysis. The wedge pixel, which was positioned at the point of a contact wedge between two grains, was the key point for separating the touching particles. Further details for searching the wedge pixels can be found in the literature [36]. Moreover, the curvature information of particle outline was added to the search procedure to optimise wedge pixels. Then, the two wedge pixels were connected to complete the separation [37].  Figure 4(d) demonstrates the effectiveness of the program to separate the touching particles marked by different colours.

**Figure 5:**
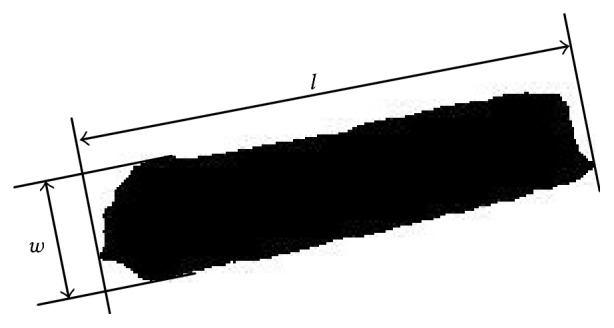
Measurement of length and width of wheat straw particle.

*Step  4*. The dimensions of particle size and shape were characterised. The maximum and minimum dimensions of the identified particle, termed as length and width, were the dominant and most significant dimensions for natural biomass particles. Once the particles were recognised, a method of dimension calculation analysed the geometry to measure the length and width. The measurement is depicted in Figure 5, where the width *w* is defined as the minimum distance between two parallel lines tangential to the projected outline of the particle, and length *l* is the distance between two tangents to the projected outline of the particle drawn perpendicularly to the tangent defining the width *w*. Particle shape had a significant effect on the probability that the particle would be classified in the correct range. In our study, wheat straw particle shape was described quantitatively by a parameter called aspect ratio (AR), which was expressed as AR = *l*/*w*. The entire program, including image processing and dimension measurement, was developed based on Matlab R2012 software.

## 3. Results and Discussion

### 3.1. PSD Analysis from Mechanical Sieving

PSD analysis is considered as a standard procedure in evaluating the morphological features of particulate materials. In view of the wider size range of wheat straw particles ground by the knife mill, mechanical sieving was conducted to divide the ground particles into several groups. In general, PSD characteristics determined through sieving analysis are expressed as a plot of mass fraction of materials retained on sieves versus screen sizes.

In this test, the wheat straw sample ground by the knife mill was divided into five groups through mechanical sieving. The mass fractions of nominal size ranges containing >1000, 500–1000, 250–500, 75–250, and <75 *μ*m were 4.18%, 13.30%, 18.77%, 37.91%, and 25.84%, respectively, where <250 *μ*m accounted for 63.75% of the total mass. Clearly, the limited quantities of screens limit the PSD sieving analysis of the particulate sample. This analysis is a collection of hundreds of noticeable dimensioned particles with the available data from four screens, thereby restricting dimensional measurement accuracy.

In addition, any particles of width less than the sieve opening could pass through the sieves regardless of particle shape. This condition illustrates the width-based separation of sieving analysis. Guo et al. [28] reported that biomass particles ground by knife mill had a needle-like shape because of the anisotropy of the material. Length served as a dominant dimension of this type of biomass material with a size range that was not determined accurately by mechanical sieving analysis. Womac et al. [21] observed this length-based separation inconsistency in particle size analysis of knife-milled ground biomass material using ASABE design sieves. Thus, in this study, classification by sieving reduced the wide size range of ground particles, which is beneficial to photograph and analyse the particle morphology.

### 3.2. Particle Morphology Analysis Based on Image Processing

Preliminary investigation on the particle imaging method demonstrated that the biomass powders, which have a nominal size of <250 *μ*m, were difficult to disperse by singulated arrangement. And these powders were too small to be easily photographed using the flatbed scanner with limited scanning resolution. Ultrasonic dispersion method and BSE imaging mode of SEM were combined to photograph the smaller particles. About 300 particles were identified randomly from pictures based on the above image processing technology to obtain detailed morphological information. After the particle dimensions were measured, the average lengths, widths, ARs, and corresponding standard deviations (SD) of ground powders in five size ranges, divided by mechanical sieving, were acquired.  Table 1 shows the average dimensional parameters of wheat straw powders in different size ranges, where the average AR is not the ratio of average length to average width but the average value of the AR of 300 particles.

As shown in Table 1, the average widths of particles are in the ranges of screen sizes, but the average lengths almost exceed the screen sizes except for the largest sieve, which does not control the upper limit of the particles. Moreover, the larger screens resulted in greater range of width, which was demonstrated by descending SD parameters. Regarding SDs, the smaller values obtained for particle dimensions corresponded to a narrower distribution. Comparing the SDs of length and width of each size range also confirmed the separation mechanism of mechanical sieving based on width.

**Figure 6:**
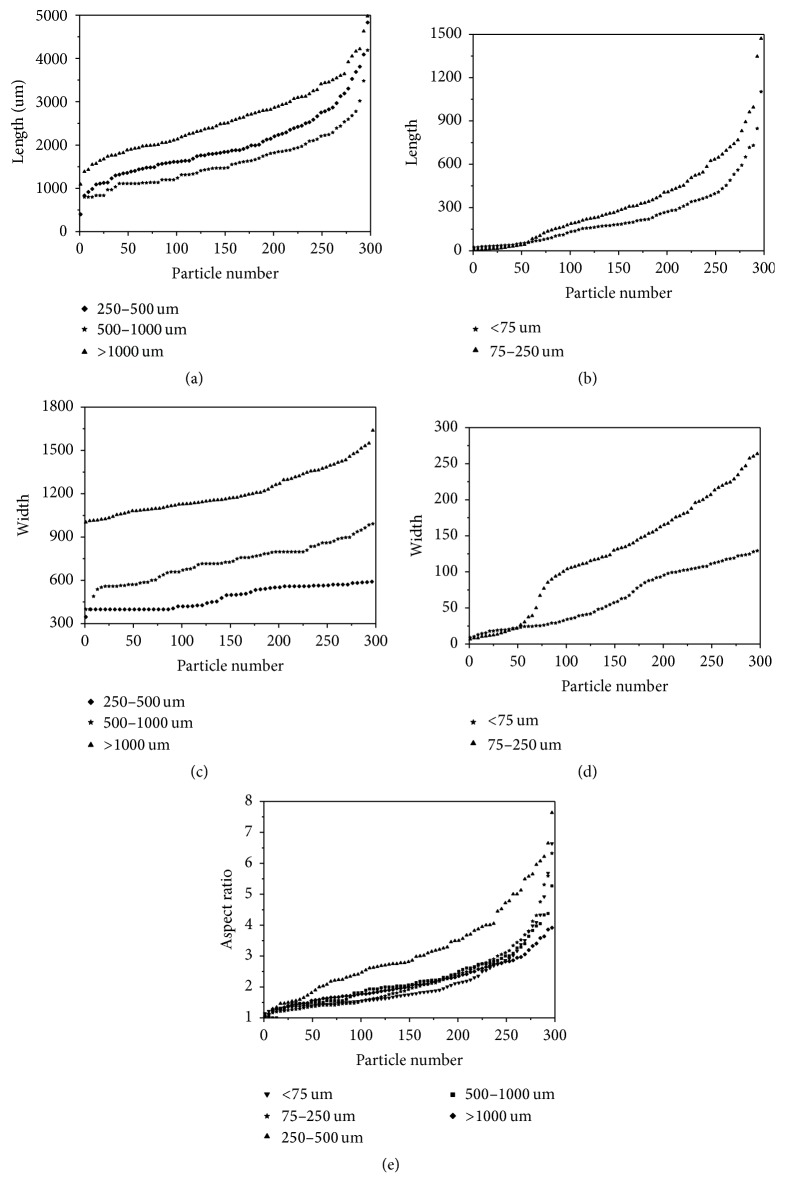
Particle size and shape distribution of ground wheat straw powders. (a) Distribution curves of lengths larger than nominal 250 *μ*m, (b) distribution curves of lengths less than nominal 250 *μ*m, (c) distribution curves of widths larger than nominal 250 *μ*m, (d) distribution curves of widths less than nominal 250 *μ*m, and (e) distribution curves of ARs.

The PSDs of 300 particles were displayed as plots of lengths, widths, and ARs sorted in ascending order versus particle number, which are shown in Figure 6. Owing to the great disparity of particle sizes in different ranges, the curves of length and width distributions were displayed separately with nominal size of 250 *μ*m as boundary. Figures 6(b) and 6(d) show the distribution curves of lengths and widths of wheat straw powders less than nominal 250 *μ*m, respectively. Based on these semilog plots, various curves corresponding to different sizes overlap each other, thereby indicating the limited efficiency of the sieving method with the current number of screens in separating the particles of various sizes.

Biomass particles obtained by size reduction processing of the knife mill were irregular because of structural anisotropy. In this study, AR was used to describe particle shape. AR is equal to one when particles are circles and squares; otherwise it is greater than one when particles are more elongated. The average ARs and SDs in Table 1 and plots of AR distribution in Figure 6(e) show that the finer particles tend to be short just as the conclusions of literature [28].

A minimum sample size for testing was needed to represent the sample well. Igathinathane et al. [38] reported that even a few grams of sample could consist of thousands of particles. An electronic analytical balance was used to ensure the accuracy of sample weight. In addition, a large number of laboratory-scale experiments were conducted to ensure statistical significance and repeatability of PSD data.

## 4. Conclusions

Particle morphological characteristics greatly influence the physical properties of granular biomass materials. A computer vision-based image processing method can be considered as an alternative for sieve analysis [22]. However, because of the resolution limit of the image acquisition unit and agglomeration of fine particles, the ground biomass powders of wide size range were difficult to identify completely.

In this study, mechanical sieving was only used to divide the sample into five groups, thereby reducing the wide size range. The powders retained on sieves, with opening sizes greater than 250 *μ*m, were photographed by a flatbed scanner without zoom function, and the others were photographed using SEM with high-image resolution. Actual imaging tests confirmed the excellent effect of BSE imaging mode of SEM.

To weaken the particle agglomeration, the singulated arrangement and ultrasonic dispersion methods were used to disperse the powders according to different size ranges. In a sense, mechanical sieving also dispersed the particles. Moreover, an image segmentation method based on geometrical information of particles, such as wedges and curvatures, was proposed to recognise the finer powders touching each other, which was the ultimate aim of this study. The distribution curves of particle size and shape obtained by experiments demonstrated that the improved image processing method can be applied to particle morphology analysis.

## Figures and Tables

**Table 1:** Average dimensional parameters of wheat straw powders in different size ranges.

Size ranges (*μ*m)	Average length (*μ*m) ± SD	Average width (*μ*m) ± SD	Average AR ± SD
>1000	2817.95 ± 799.82	1214.96 ± 155.38	2.38 ± 1.89
500–1000	1479.89 ± 674.65	637.52 ± 133.62	2.25 ± 1.47
250–500	797.40 ± 807.23	367.01 ± 75.46	2.19 ± 1.32
75–250	280.83 ± 327.73	143.34 ± 75.15	2.12 ± 1.15
<75	65.14 ± 223.78	34.86 ± 39.14	1.91 ± 1.09
